# Mesenchymal stem cell-derived conditioned medium protects vascular grafts of brain-dead rats against in vitro ischemia/reperfusion injury

**DOI:** 10.1186/s13287-021-02166-3

**Published:** 2021-02-24

**Authors:** Sevil Korkmaz-Icöz, Pengyu Zhou, Yuxing Guo, Sivakkanan Loganathan, Paige Brlecic, Tamás Radovits, Alex Ali Sayour, Mihály Ruppert, Gábor Veres, Matthias Karck, Gábor Szabó

**Affiliations:** 1 Department of Cardiac Surgery, Laboratory of Cardiac Surgery, University Hospital Heidelberg, INF 326, 69120 Heidelberg, Germany; 2 Department of Cardiac Surgery, University Hospital Halle (Saale), Halle, 06120 Germany; 3 Heart and Vascular Center, Semmelweis University, Budapest, 1122 Hungary

**Keywords:** Ischemia/reperfusion, Endothelial function, Mesenchymal stem cells, Conditioned medium, Brain death

## Abstract

**Background:**

Brain death (BD) has been suggested to induce coronary endothelial dysfunction. Ischemia/reperfusion (IR) injury during heart transplantation may lead to further damage of the endothelium. Previous studies have shown protective effects of conditioned medium (CM) from bone marrow-derived mesenchymal stem cells (MSCs) against IR injury. We hypothesized that physiological saline-supplemented CM protects BD rats’ vascular grafts from IR injury.

**Methods:**

The CM from rat MSCs, used for conservation purposes, indicates the presence of 23 factors involved in apoptosis, inflammation, and oxidative stress. BD was induced by an intracranial-balloon. Controls were subjected to a sham operation. After 5.5 h, arterial pressures were measured in vivo. Aortic rings from BD rats were harvested and immediately mounted in organ bath chambers (BD group, *n* = 7) or preserved for 24 h in 4 °C saline-supplemented either with a vehicle (BD-IR group, *n* = 8) or CM (BD-IR+CM group, *n* = 8), prior to mounting. Vascular function was measured in vitro. Furthermore, immunohistochemistry and quantitative real-time polymerase chain reaction (qRT-PCR) have been performed.

**Results:**

BD in donors was associated with significantly impaired hemodynamic parameters and higher immunoreactivity of aortic myeloperoxidase (MPO), nitrotyrosine, caspase-3, caspase-8, caspase-9, and caspase-12 compared to sham-operated rats. In organ bath experiments, impaired endothelium-dependent vasorelaxation to acetylcholine in the BD-IR group compared to BD rats was significantly improved by CM (maximum relaxation to acetylcholine: BD 81 ± 2% vs. BD-IR 50 ± 3% vs. BD-IR + CM 72 ± 2%, *p* < 0.05). Additionally, the preservation of BD-IR aortic rings with CM significantly lowered MPO, caspase-3, caspase-8, and caspase-9 immunoreactivity compared with the BD-IR group. Furthermore, increased mRNA expression of vascular cell adhesion molecule (VCAM)-1 and intercellular adhesion molecule (ICAM)-1 in the aortas from the BD-IR rats compared to BD group were significantly decreased by CM.

**Conclusions:**

The preservation of BD rats’ vascular grafts with CM alleviates endothelial dysfunction following IR injury, in part, by reducing levels of inflammatory response and caspase-mediated apoptosis.

**Supplementary Information:**

The online version contains supplementary material available at 10.1186/s13287-021-02166-3.

## Introduction

Heart transplantation remains the therapy of choice for patients with refractory heart failure [1]. Currently, hearts are mainly procured from brain-dead (BD) donors. However, coronary endothelial dysfunction is well-described after cardiac transplantation [2] and has been also suggested following brain death [3]. Usually, brain death is a consequence of increased intracranial pressure, which initially causes compensatory arterial hypertension to maintain adequate cerebral perfusion pressure, tachycardia, and intense peripheral vasoconstriction due to an early catecholamine storm [4]. After this hypertensive phase, there is a loss of sympathetic tone and subsequent peripheral vasodilation, resulting in hypotension [5]. Furthermore, brain death triggers inflammatory response. In heart transplantation, ischemia/reperfusion (IR) injury is a major issue. IR-induced disturbance of the endothelium’s functional integrity, known as “endothelial dysfunction,” is caused by the rapid normalization of the acidotic pH following reperfusion, which initiates the depletion of high-energy phosphates [6], high production of reactive oxygen species (ROS), the generation of inflammatory cytokines and infiltrating neutrophils [7], and finally leads to cell death [8]. The role of acidosis was evidenced by ischemic acidosis-induced apoptosis in coronary endothelial cells of arteries through activation of caspase-12 and caspase-3 [9]. Thus, any pre-existent vascular damage due to brain death may be aggravated by hypothermic preservation/warm reperfusion during transplantation. Taken together, the significance of vascular endothelial dysfunction makes the endothelium an attractive target during the perioperative management of heart transplantation. However, endothelial structural and functional integrity under conditions of organ preservation following brain death have not been extensively investigated [10]. Therefore, further studies are warranted to identify novel strategies to prevent adverse effects of IR injury and brain death, not only for cardiomyocytes but also the endothelium, so that endothelial protection could be shifted into cardiac protection.

Bone marrow-derived mesenchymal stem cells (MSCs), multipotent and self-renewing cells [11], are emerging as a promising cell-based therapy for IR-induced myocardial infarction [12, 13]. The potential therapeutic effects of MSCs have been attributed to their excellent properties in immunomodulation, angiogenesis, the capacity to differentiate into multiple tissue types, the ability to engraft into injured tissue, and direct cell-to-cell contact [14]. However, recent studies have revealed that the long-term survival rate of implanted MSCs is relatively low [15, 16], suggesting an alternative mode of repair, including paracrine mechanisms [17]. An increasing number of literature brought attention to the vast array of factors produced by MSCs, such as chemokines/cytokines [18] and anti-apoptotic and growth factors, which could play a significant protective role [19, 20]. A previous ex vivo study reported that conditioned medium (CM) from bone marrow-derived MSCs added at the onset of reperfusion following ischemia conferred protection against myocardial IR injury [21]. The protection induced by CM included attenuation of apoptosis and oxidative stress in the heart [22]. We have shown that the presence of CM in preservation solution during prolonged ischemic time improves functional post-transplant graft recovery in 15-month-old rats [23]. Furthermore, our recent in vivo study provides experimental evidence that the preservation of BD donor hearts with cardioplegic solution enriched with CM improves graft contractility after heart transplantation [24].

Taken together, we hypothesized that physiological saline solution-supplemented CM protects vascular grafts of BD rats from IR injury. Next, we explored the potential molecular mechanisms underlying the beneficial effect of CM on endothelial dysfunction due to both IR injury and brain death. We investigated its link to nitro-oxidative stress, inflammation, and apoptosis.

## Materials and methods

See the Online Appendix for further details.

### Animals

Inbred male Lewis rats (8–12 weeks old; Janvier Labs, Saint Berthevin, France) received care in compliance with the Guide for the Care and Use of Laboratory Animals (National Institutes of Health Publication No. 85-23, revised 1996). All procedures and handling of animals during the investigations were reviewed and approved by the Ethical Committee of the Regional Council of Karlsruhe, Land Baden-Württemberg for Animal Experimentation (G37/14). The animals were housed at constant ambient temperature (22 ± 2 °C) in light-controlled rooms (12-12 h light-dark cycles), were given food and water access ad libitum, and were acclimatized for 1 week.

### Preparation of bone marrow-derived MSCs-CM

**Fig. 1:**
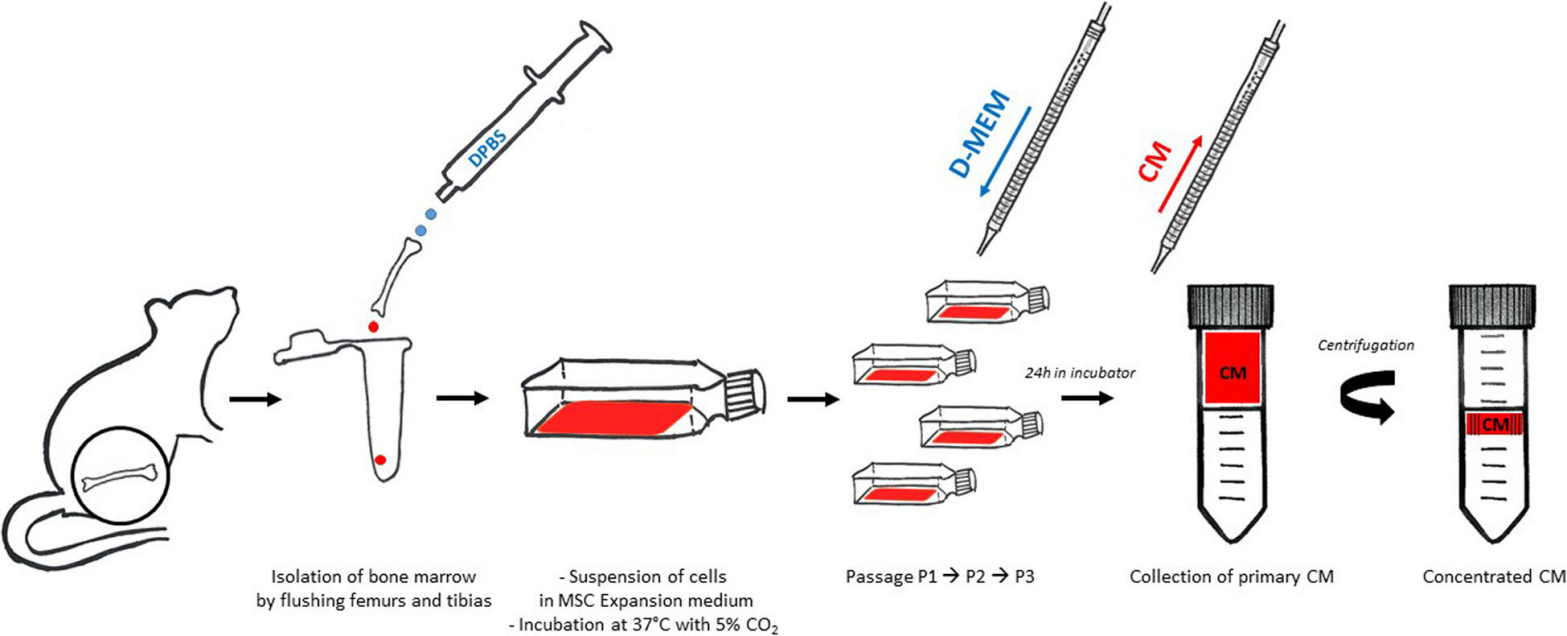
A simplified schematic of the bone marrow-derived mesenchymal stem cell (MSC)-conditioned medium (CM) collection protocol. Rats were euthanized with an overdose of pentobarbital sodium (100 mg/kg, intraperitoneally). The bone marrow was isolated by flushing the femurs and tibias with Dulbecco’s phosphate-buffered saline (DPBS). The cells were suspended in MSC Expansion Medium and then incubated at 37 °C with 5% CO_2_ on cell culture flasks. Primary cells were subcultured 1:3 when 80% confluency was reached. After MSCs reached greater than 80% confluency at passage 3, the medium was aspirated, and MSCs were rinsed 3 times with DPBS. Then, Dulbecco’s modified Eagle’s medium (D-MEM) was added to culture dishes with MSCs, and the culture dishes were put into an incubator for 24 h. Primary CM was collected and centrifuged by ultrafiltration units (4500*g* for 4 h at 4 °C) to yield concentrated CM

As previously reported [25], rats were euthanized with an overdose of pentobarbital sodium (100 mg/kg, intraperitoneally). The bone marrow was isolated by flushing the femurs and tibias with Dulbecco’s phosphate-buffered saline (DPBS) (Sigma, St. Louis, MO, USA). The cells were suspended in MSC Expansion Medium (R&D System, Minneapolis, MN, USA) and then incubated at 37 °C with 5% CO_2_ on cell culture flasks. Primary cells were subcultured 1:3 when 80% confluency was reached. After MSCs reached greater than 80% confluency at passage 3, the medium was aspirated, and MSCs were rinsed 3 times with DPBS. Then, Dulbecco’s modified Eagle’s medium (D-MEM) (Life Technologies, Grand Island, NY, USA) was added to culture dishes with MSCs, and the culture dishes were put into an incubator for 24 h. Primary CM was collected and centrifuged by ultrafiltration units (4500*g* for 4 h at 4 °C) to yield concentrated CM. A simplified schematic of the MSCs-CM collection protocol is shown in Fig. 1. The protein concentration of the CM was quantified by *Bradford protein assay* to ensure that equal concentrations (0.5 mg/mL) of CM was used. D-MEM was regarded as a control (nonconditioned medium).

### Screening of secreted proteins in CM

The cytokines/chemokines of isolated CM were qualitatively measured by using Rat Antibody Array 90 (RayBiotech, Norcross, GA).

### Model of brain death

As previously reported [26, 27], the rats were anesthetized with pentobarbital sodium (60 mg/kg, intraperitoneally), then intubated and ventilated by a rodent respirator (Harvard Apparatus, Holliston, MA, USA). The right carotid artery and left external jugular vein were cannulated for pressure monitoring (Micromanometer catheter, Millar Instruments, Houston, TX, USA) and ringer infusion, respectively. The body temperature (measured via a rectal probe) was maintained between 36.5 and 37.5 °C with a heat mat. A small 4F burr hole was drilled into the parietal skull, and a balloon catheter was introduced subdurally and inflated with 15 μL of saline per minute until a total volume of 600 μL was reached. The state of brain death was confirmed by the sustained absence of spontaneous breathing and brainstem reflexes. After reaching the final volume, the blood pressure was stabilized. A drop of blood pressure < 60 mmHg was avoided by volume administration using ringer solution without inotropic or vasoactive agents. Control donor rats were subjected to a sham operation. Sham-operated and BD donors underwent continuous hemodynamic monitoring for 5.5 h.

### Model of vascular dysfunction induced by ischemic storage and reperfusion

#### Preparation of isolated thoracic aortic rings

At 5.5 h post-sham operation or brain death, the thoracic aorta was explanted and immediately placed in cold (4 °C) Krebs-Henseleit solution (KHL) (118 mM NaCl, 4.7 mM KCl, 1.2 mM KH_2_PO_4_, 1.2 mM MgSO_4_, 1.77 mM CaCl_2_, 25 mM NaHCO_3_, 11.4 mM glucose; pH 7.4) and treated with 95% O_2_–5% CO_2_. After careful dissection of adhering fat and connective tissue without damaging the endothelium, the aorta was cut into several segments (each 4 mm in length).

#### Experimental groups

As previously reported [28], physiological saline was treated with nitrogen to extrude oxygen from the solution. Then, the aortic rings were placed in closed, air-free tubes filled with saline supplemented with either D-MEM vehicle [BD-IR (*n* = 8 rats, 32 rings) group] or CM [BD-IR+CM (*n* = 8 rats, 32 rings) group] and stored for 24 h at 4 °C. After cold ischemic conservation, the rings were mounted in organ bath chambers. To simulate free radical burst and endothelial dysfunction, which usually occurs during reperfusion in vivo, hypochlorite was added to the baths (200 μM, 30 min). Aortic rings in the sham (*n* = 7 rats, 24 rings) and BD (*n* = 8 rats, 26 rings) normoxia groups did not undergo cold ischemic storage but were immediately mounted in organ baths after their preparation.

#### In vitro organ Bath experiment

Aortic rings were mounted on stainless steel hooks in organ baths (Emka Technologies, Paris, France), containing 20 mL of KHL and continuously treated with 95% O_2_–5% CO_2_ at 37 °C. The rings were placed under an optimal resting tension of 2 g for 60 min for equilibration. During this period, the tension was cyclically adjusted to the 2 g resting tension, and KHL was replaced every 30 min. After the equilibration period, the stretched rings were contracted with potassium chloride (KCl, 80 mM) to obtain maximal contraction forces. Then, the aortic rings were washed to acquire resting tension again. Relaxation responses were examined with the endothelium-dependent vasorelaxant acetylcholine (ACh) and the endothelium-independent vasorelaxant agent sodium nitroprusside (SNP). Briefly, aortic rings were precontracted with phenylephrine (PE, 10^−9^–10^−5^ M) until a stable plateau was reached. Relaxation responses were obtained by the addition of cumulative concentrations of ACh (10^−9^–10^−4^ M). Then, the aortic rings were washed to obtain resting tension, and PE (10^−6^ M) was added to the organ baths until a new, stable plateau was reached. At last, SNP (10^−10^–5 × 10^−7^ M) was applied to test the relaxation response of smooth muscle cells. The tension was recorded with isometric force transducers of a myograph (159901A; Radnoti Glass Technology, Monrovia, CA, USA), digitized, and displayed with the IOX Software System (EMKA Technologies, Paris, France). Contractile responses to KCl were described in grams of tension, and contractile responses to PE were expressed as a percent of the maximum contraction induced by KCl. By fitting experimental data to a sigmoidal equation using Origin 7.0 (Microcal Software Northampton, MA, USA), half-maximum response (EC_50_) values were generated from individual concentration responses. The sensitivity to PE, ACh, and SNP was assessed by negative logarithm of the corresponding EC_50_ (pD_2_ = −log EC_50_ (M)), and vasorelaxation (and its maximum) was expressed as the percentage of contraction induced by phenylephrine.

### Immunohistochemistry

The immunoreactivity to caspase-3 (1:400; Novus Biologicals, Littleton, CO), caspase-8 (1:1000; Novus Biologicals, Littleton, CO), caspase-9 (1:50; Santa Cruz Biotechnology, Dallas, TX), caspase-12 (1:200; Novus Biologicals, Littleton, CO), myeloperoxidase (MPO) (1:200; Abcam, Cambridge, UK), and nitrotyrosine (1:200; MilliporeSigma, Burlington, MA) was assessed. Infiltrating neutrophils (MPO-labeled) were counted; caspase-3, caspase-8, caspase-9, caspase-12, and nitrotyrosine expression were semi-quantitatively assessed based on staining intensity and the distribution of the labeled target protein. The analysis was performed under a conventional light microscope in a blinded fashion. The intensity score values were given as follows: 0, no positive staining; 1, weak staining; 2, intermediate staining; and 3, extensive staining, and an area score was assigned as follows: 1 = up to 10% positive cells, 2 = 11–50% positive cells, 3 = 51–80% positive cells, and 4 = more than 80% positive cells, using the ImageJ analysis system (NIH, MD, USA). The total score of each field was calculated as intensity score multiplied by area score (0–12). The evaluation was carried out in four random and nonoverlapping fields of the aorta, and the average value was calculated for each animal.

### Quantitative real-time polymerase chain reaction

As previously reported [29, 30], the total RNA was isolated with the RNeasy Fibrous Tissue Mini Kit (Qiagen, Hilden, Germany). The RNA concentration and purity were determined by absorbance measurements at 230 nm, 260 nm, and 280 nm. cDNA synthesis was performed with the QuantiTect Reverse Transcription Kit (Qiagen, Hilden, Germany) using 250 ng of isolated total RNA in a total volume of 20 μL. Quantitative real-time PCR was performed with the Light-Cycler480 system combining LightCycler480 Probes Master with Universal ProbeLibrary probes (Roche, Mannheim, Germany) (Online Table 1). The conditions for the qRT-PCR were as follows: 95 °C for 10 min (1 cycle), 95 °C for 10 s, 60 °C for 30 s (single; 45-cycle quantification), and 40 °C for 10 s (1 cycle). The reaction volume was 20 μL. The following targets were investigated: caspase-3, intercellular adhesion molecule (ICAM)-1 and vascular cell adhesion molecule (VCAM)-1. Sample quantifications were normalized to glyceraldehyde 3-phosphate dehydrogenase (GAPDH) expression, by using a pool of all cDNAs (positive calibrator). The evaluation was performed with the Light Cycler 480 SW 1.5 software (Roche, Mannheim, Germany).

### Statistical analysis

The results were expressed as mean ± standard error of the mean (SEM). Statistical analysis was performed using the GraphPad Prism 7.02 software (GraphPad Software, Inc., CA). Data was tested for normality using the Shapiro-Wilk test before statistical tests. The two-sample student *t* test was used to compare the means of sham and BD groups from normally distributed data, and a nonparametric Mann-Whitney test was applied if data were not normally distributed. In all other cases, one-way ANOVA followed by Tukey’s post hoc test was applied for multiple comparisons. If the data were not normally distributed, the nonparametric Kruskal-Wallis test followed by Dunn’s post hoc test was used. A value of *p* < 0.05 was considered statistically significant.

## Results

### Characterization of BD rat model

#### Effect of brain death on hemodynamic parameters

Heart rate (342 ± 6 vs. 395 ± 8 beats/min, *p* < 0.05), systolic blood pressure (63 ± 1 vs. 126 ± 4 mmHg, *p* < 0.05), diastolic blood pressure (41 ± 2 vs. 94 ± 4 mmHg, *p* < 0.05), mean arterial pressure (48 ± 1 vs. 105 ± 4 mmHg, *p* < 0.05), and pulse pressure (22 ± 1 vs. 32 ± 2 mmHg, *p* < 0.05) were significantly decreased in the BD group compared to the sham-operated rats.

#### Effect of brain death on contractile and relaxant responses

**Fig. 2:**
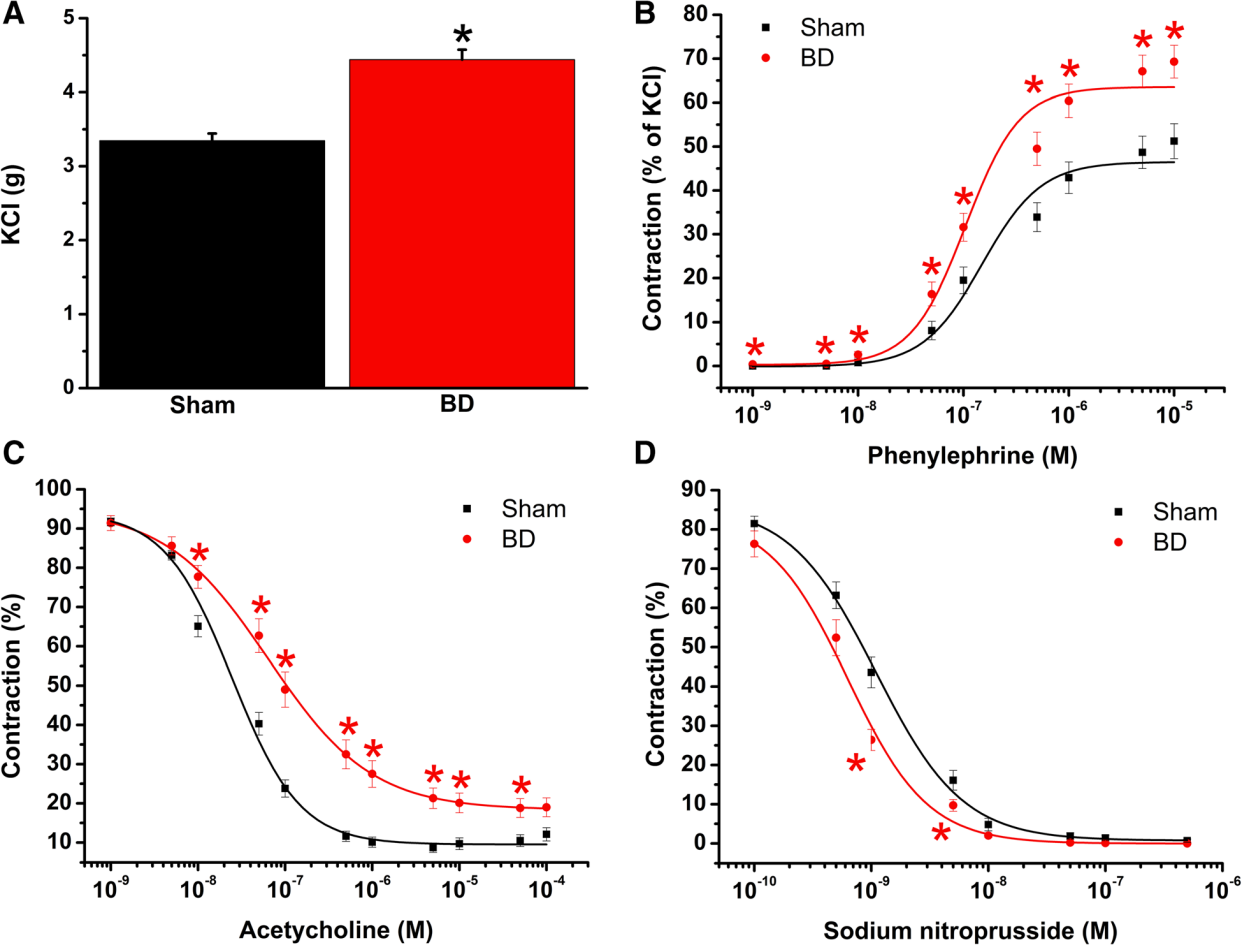
Effect of brain death (BD) on contractile and relaxant responses. Contractile responses (**a**) to high K^+^-induced depolarization and **b** for phenylephrine (percentage of the maximum contraction induced by potassium chloride (KCl)), and **c** acetylcholine-induced endothelium-dependent and **d** sodium nitroprusside-induced endothelium-independent vasorelaxation of isolated aortic rings. Values are mean ± SEM. *p < 0.05 vs. sham. *n* = 24–26 rings from 6 to 7 rats

To examine the effects of brain injury on vascular functional changes, isolated organ bath technique was used. Compared with the sham-operated group, both maximum contractile responses to high K^+^-induced depolarization and adrenergic alpha-1 agonist PE were significantly higher in the BD group (Table 1, Fig. 2a, b). ACh induced a concentration-dependent relaxation in aortic rings precontracted with PE (Fig. 2c). Maximal endothelium-dependent relaxation (*R*_max_) to ACh was significantly impaired in the aortic rings in the BD group compared with the sham-operated animals. Moreover, brain death significantly decreased aortic sensitivity (pD_2_ value) to ACh (Table 1). Although maximum endothelium-independent vasorelaxation to SNP did not differ between the two groups, the concentration-response curve from BD aortas tended to be left-shifted compared to sham-operated rats (Table 1, Fig. 2d).

**Table 1:** Quantitative analysis of vascular function

	KCl (g)	PE (% of KCl)	pD_**2**_ to PE	***R***_**max**_ to ACh (%)	pD_**2**_ to ACh	***R***_**max**_ to SNP (%)	pD_**2**_ to SNP
BD	4.4 ± 0.1	69 ± 4	6.8 ± 0.1	81 ± 2	7.1 ± 0.1	100 ± 0	9.4 ± 0.1
BD-IR	2.7 ± 0.2*	132 ± 10*	7.1 ± 0.1*	50 ± 3*	6.7 ± 0.1	100 ± 0	9.1 ± 0.1
BD-IR+CM	2.8 ± 0.1*	91 ± 6*^,#^	6.6 ± 0.1^#^	72 ± 2^#^	7.3 ± 0.1^#^	99 ± 0	9.3 ± 0.1

*KCl* potassium chloride, *PE* phenylephrine, *ACh* acetylcholine, *SNP* sodium nitroprusside, *IR* ischemia/reperfusion, *CM* conditioned medium, *BD* brain death, *R*_*max*_ maximum relaxation, *pD*_*2*_*, -logEC*_*50*_ EC_50_ being the concentration of substance that elicited 50% of the maximum response. Values are means ± SEM

*p < 0.05 vs. BD, ^#^*p* < 0.05 vs. BD-IR

*n* = 26–34 rings from 7 to 9 rats

#### Effect of brain death on neutrophil infiltration, nitro-oxidative stress, and apoptosis

**Fig. 3:**
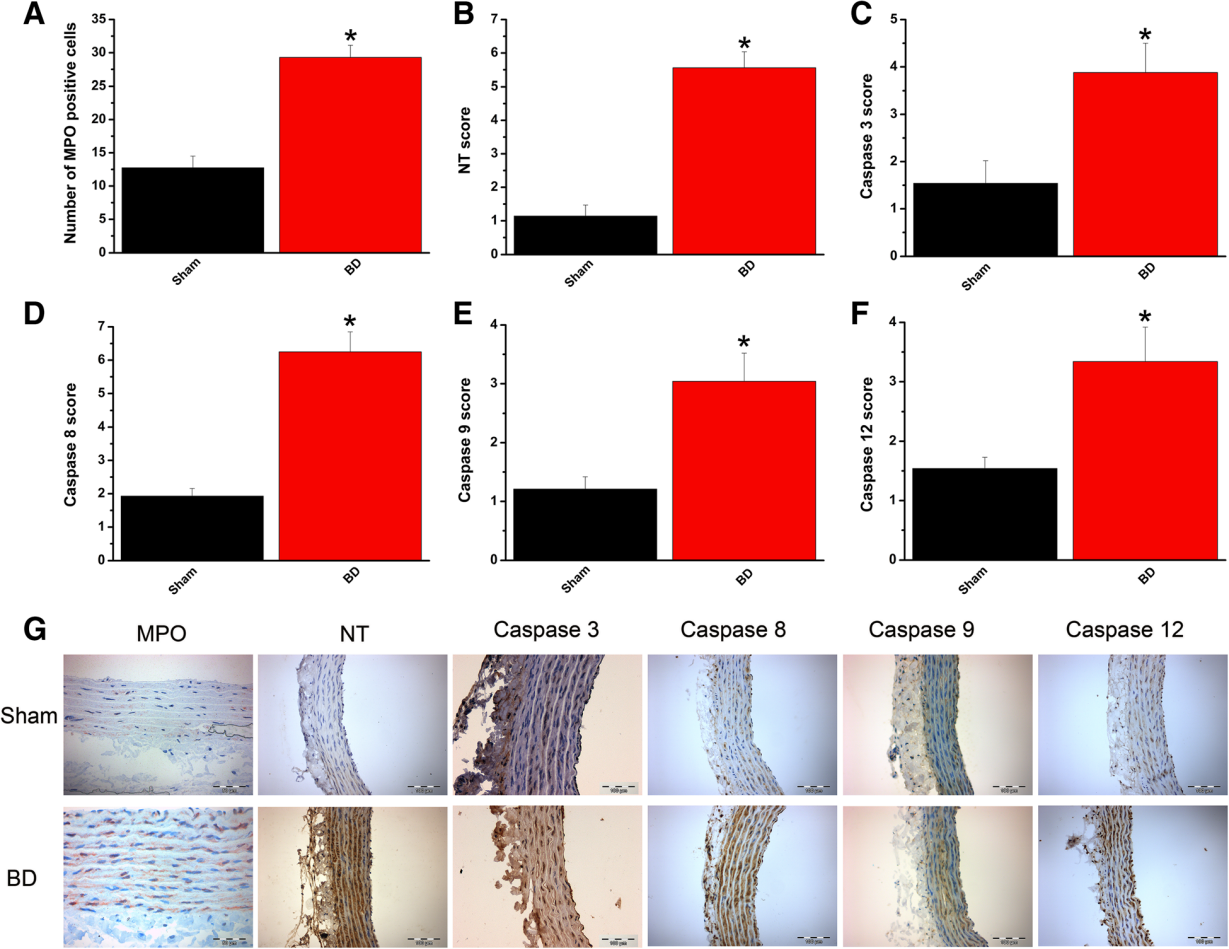
Effect of brain death (BD) on neutrophil infiltration, apoptosis, and nitro-oxidative stress. Immunohistochemical scores of stainings for **a** myeloperoxidase (MPO, × 400, scale 50 μm), **b** nitrotyrosine (NT, × 200, scale 100 μm), **c** caspase-3 (× 200, scale 100 μm), **d** caspase-8 (× 200, scale 100 μm), **e** caspase-9 (× 200, scale 100 μm), and **e** caspase-12 (× 200, scale 100 μm). **f** Representative photomicrographs. Values are mean ± SEM. *p < 0.05 vs. sham. *n* = 6–8 rats

To determine whether brain death induction was associated with cellular changes in the aorta, we performed immunohistochemistry. Neutrophil infiltration, assessed by staining for MPO, was significantly higher in BD rings compared to the sham-operated rats (Fig. 3a). Detection of nitrotyrosine is regarded as a marker of nitro-oxidative stress. Intense nitrotyrosine immunoreactivity was present in the BD group compared to controls (Fig. 3b). Analysis of apoptosis in aortic tissue also revealed that caspase-3, caspase-8, caspase-9, and caspase-12 immunoreactivity was significantly higher after brain injury compared to the sham-operated animals (Fig. 3c–f).

### Effect of CM against IR injury in BD rats’ aorta

#### Characterization of the CM

**Fig. 4:**
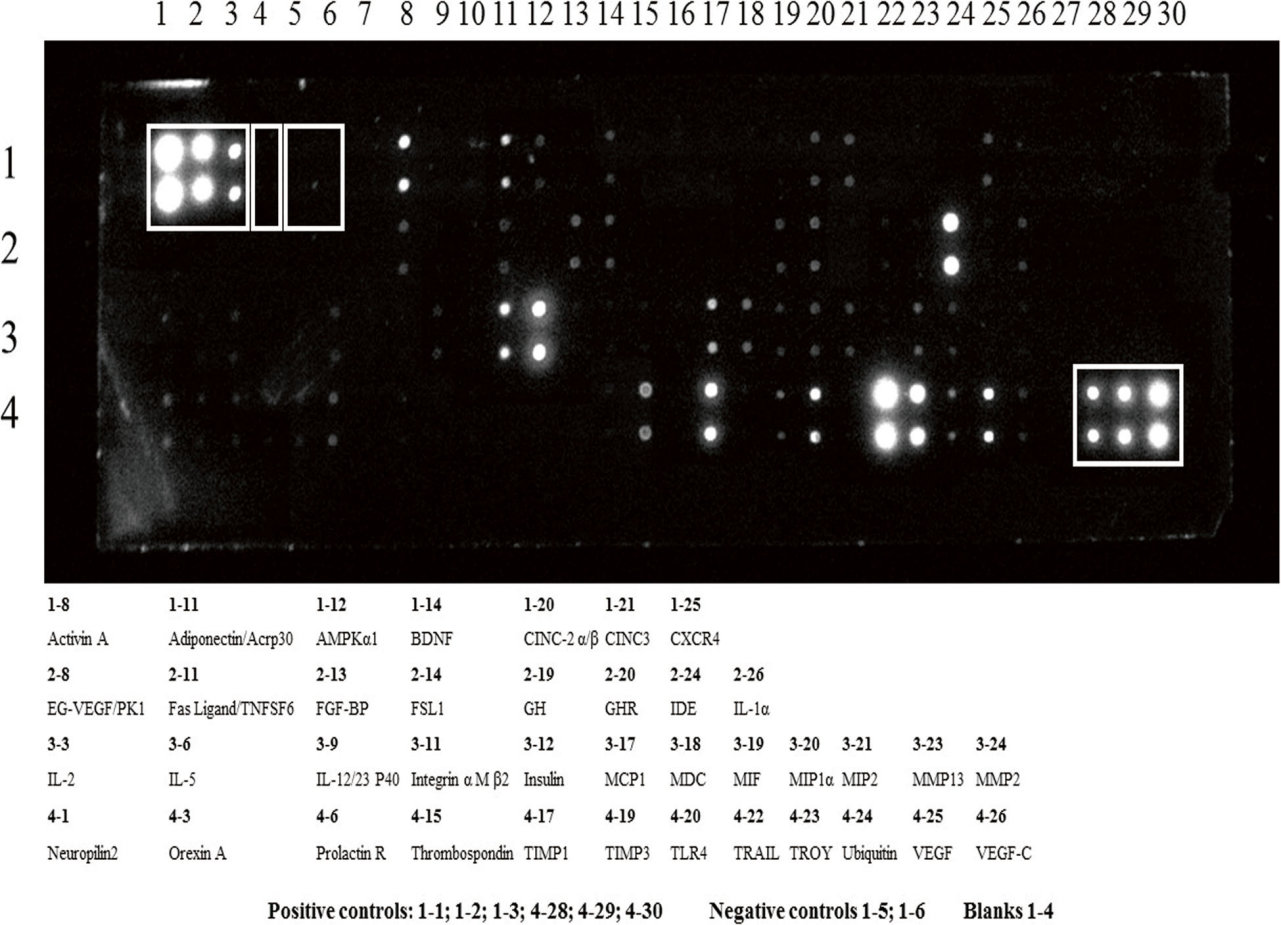
Characterization of bone marrow-derived mesenchymal stem cell-conditioned medium by an antibody array. In the upper left (1–1, 1–2, and 1–3) and lower right (4–28, 4–29, and 4–30) corners, the squares correspond to positive control signals (equal amounts of biotinylated immunoglobulin G printed directly onto the array). The squares in 1–5 and 1–6 correspond to negative control spots (protein-containing buffer), and 1–4 is blank spots (meaning that nothing is printed here)

The characterization of soluble factors secreted by MSCs with antibody-based protein array analysis revealed that CM contained 39 of 90 proteins, including cytokines/chemokines, growth factors, adhesion molecules, and other proteins. Among them, at least 23 factors are involved in apoptosis, inflammation, and oxidative stress (Table 2, Fig. 4).

**Table 2:** List of 23 factors involved in apoptosis, inflammation, and oxidative stress in conditioned medium

Properties	Factors
Anti-apoptotic	TIMP-1, growth hormone, growth hormone receptor, EG-VEGF (PK1), VEGF, VEGF-C, activin A, BDNF, FGF-BP
Pro-apoptotic	TRAIL, thrombospondin, TROY, Fas ligand/TNFSF6
Anti-inflammatory	CXCR-4, MDC
Pro-inflammatory	CINC-2, CINC-3, FSL-1, MCP-1, MDC, MIF, MIP-1α, MMP-13, TLR4, Fas ligand/TNFSF6, CXCR-4
Protection from oxidative stress	FGF-BP

*TIMP-1* tissue inhibitors of metalloproteinases-1, *EG-VEGF* endocrine gland-derived vascular endothelial growth factor, *PK1* prokineticin-1, *VEGF* vascular endothelial growth factor, *VEGF-C* vascular endothelial growth factor-C, *BDNF* brain-derived neurotrophic factor, *FGF-BP* fibroblast growth factor-binding protein, *TRAIL* tumor necrosis factor-related apoptosis-inducing ligand, *TROY* tumor necrosis factor receptor superfamily, member-19, *TNFSF6* tumor necrosis factor superfamily, member-6, *CXCR-4* C-X-C chemokine receptor type-4, *MDC* macrophage-derived chemokine, *CINC* cytokine-induced neutrophil chemoattractant, *FSL-1* follostatin-like-1, *MCP-1* monocyte chemoattractant protein-1, *MIF* macrophage migration inhibitory factor, *MIP-1α* macrophage inflammatory proteins 1-alpha, *MMP-13* matrix metalloproteinase-13, *TLR4* Toll-like receptor-4

#### Effect of CM on contractile responses after IR injury

**Fig. 5:**
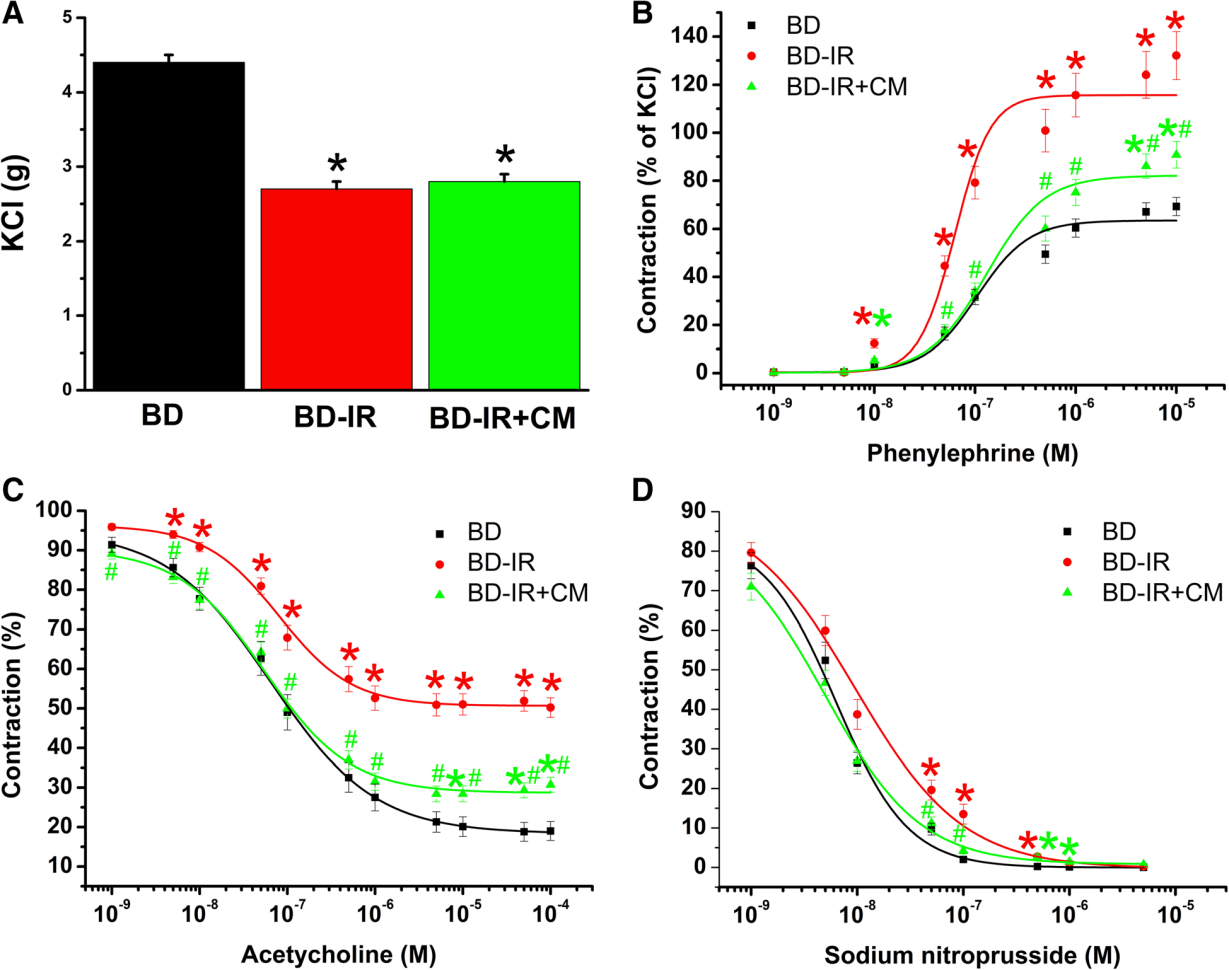
Effect of conditioned medium on contractile and relaxation responses after ischemia/reperfusion injury. Contractile responses **a** to high K^+^-induced depolarization and **b** for phenylephrine (percentage of the maximum contraction induced by potassium chloride (KCl)). **c** Acetylcholine-induced endothelium-dependent and **d** sodium nitroprusside-induced endothelium-independent vasorelaxation of isolated aortic rings. CM, conditioned medium; IR, ischemia/reperfusion; BD, brain death. Values are mean ± SEM. *p < 0.05 vs. BD and ^#^*p* < 0.05 vs. BD-IR. *n* = 24–34 rings from 7 to 9 rats

Contractile responses to KCl were significantly impaired in the BD-IR group compared to BD rats, and CM had no effect (Table 2, Fig. 5a). Higher contractile responses to PE observed in the BD-IR aortas compared to the BD group were significantly reduced by CM (Table 1, Fig. 5b). Additionally, increased aortic ring sensitivity (pD_2_ value) to PE seen after IR injury in the BD group was significantly decreased by CM (Table 1).

#### Effect of CM on endothelium-dependent vasorelaxation after IR injury

Decreased *R*_max_ to ACh in the BD-IR group compared to BD rats was significantly improved by the preservation of the aortic rings with CM, indicating an amelioration of endothelial function (Table 2, Fig. 5c). Additionally, decreased aortic ring sensitivity to ACh (as expressed in pD_2_ value) seen after IR injury was significantly increased by CM (Table 1).

#### Effect of CM on endothelium-independent vasorelaxation after IR injury

Figure 4d shows the concentration-dependent vasorelaxation induced by SNP, an endothelium-independent vasodilator. Although maximal vasorelaxation to SNP did not differ among the experimental groups, the concentration-response curve in the aortas from BD-IR+CM tended to be left-shifted compared to the BD-IR group (Table 2, Fig. 5d).

#### Effect of CM on neutrophil infiltration, nitro-oxidative stress, and apoptosis after IR injury

**Fig. 6:**
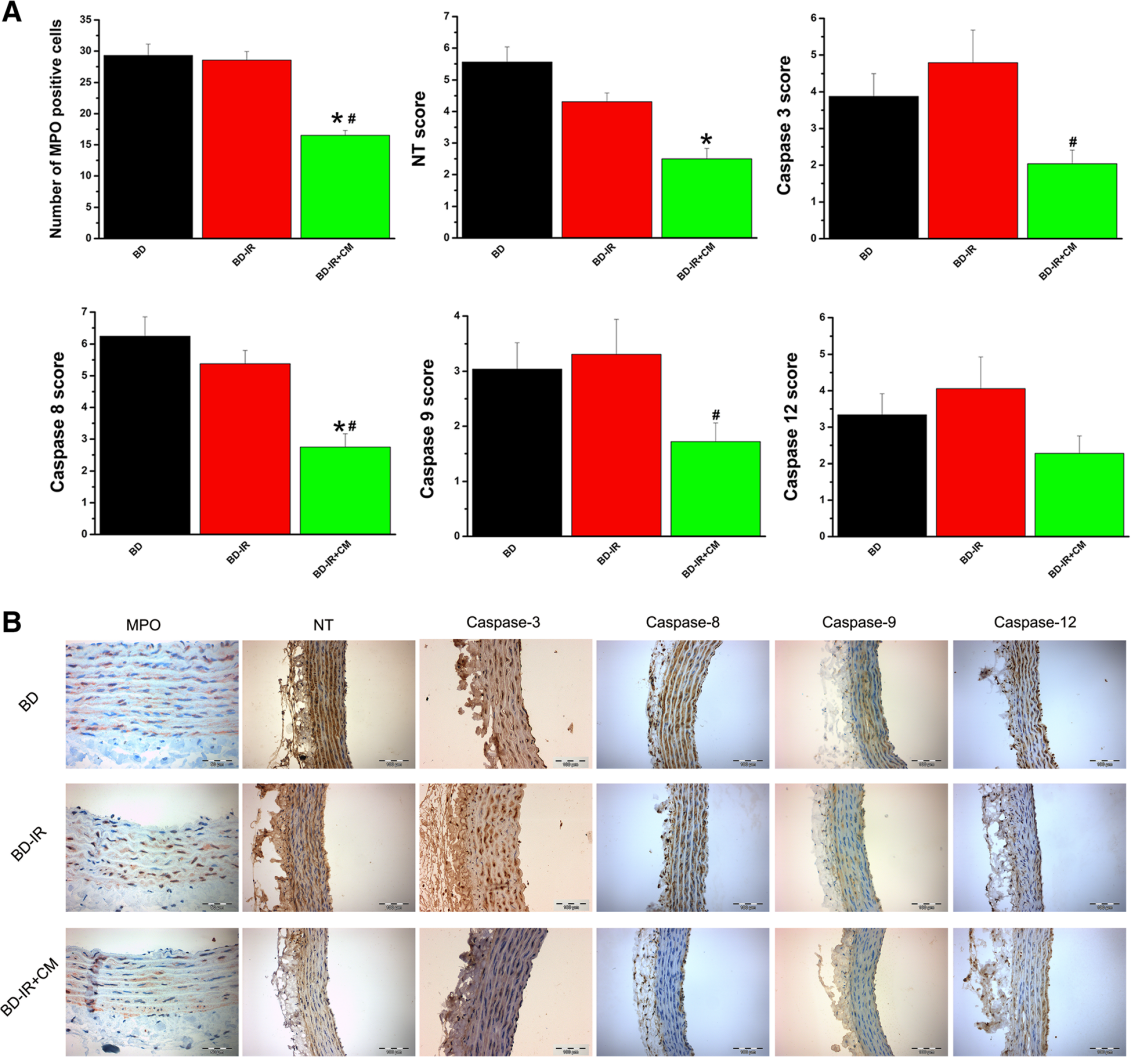
Effect of conditioned medium on neutrophil infiltration, apoptosis, and nitro-oxidative stress after ischemia/reperfusion injury. **a** Immunohistochemical scores of stainings for myeloperoxidase (MPO, × 400, scale 50 μm), nitrotyrosine (NT, × 200, scale 100 μm), caspase-3 (× 200, scale 100 μm), caspase-8 (× 200, scale 100 μm), caspase-9 (× 200, scale 100 μm), and caspase-12 (× 200, scale 100 μm). **b** Representative photomicrographs. CM, conditioned medium; IR, ischemia/reperfusion; BD, brain death. Values are mean ± SEM. *p < 0.05 vs. BD and ^#^*p* < 0.05 vs. BD-IR. *n* = 6–9 rats

Immunohistochemical data showed that assessment of neutrophil infiltration by quantification of MPO-positive cell infiltration and immunoreactivity for nitrotyrosine, caspase-3, caspase-8, caspase-9, and caspase-12 with brown staining did not further increase in the aortic rings from the BD-IR group compared to the BD rats (Fig. 6). However, the preservation of IR aortic rings with CM significantly lowered MPO-positive neutrophil infiltration and caspase-3, caspase-8, and caspase-9 immunoreactivity compared with the BD-IR group and had a tendency toward lower nitrotyrosine and caspase-12 positivity (Fig. 6).

#### Effect of CM on gene expression changes after IR injury

**Fig. 7:**
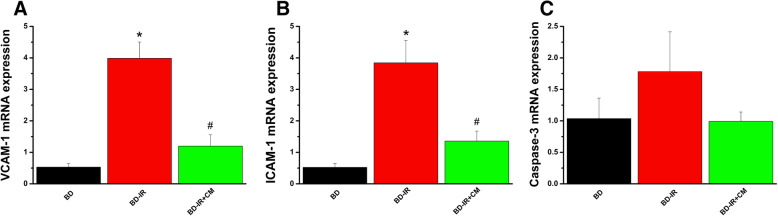
Effect of conditioned medium on gene expression changes after ischemia/reperfusion injury. **a** Vascular cell adhesion molecule (VCAM)-1, **b** intercellular adhesion molecule (ICAM)-1, and **c** caspase-3. CM, conditioned medium; IR, ischemia/reperfusion; BD, brain death. Values are mean ± SEM. *p < 0.05 vs. BD and ^#^*p* < 0.05 vs. BD-IR. *n* = 3–6 rats

qRT-PCR revealed an increase in the gene expression of VCAM-1 and ICAM-1 in the BD-IR group compared to BD rats, which was significantly decreased by the preservation of aortic rings with CM (Fig. 7a, b). We observed a trend for increased caspase-3 expression in the BD-IR group compared to BD and a decrease after CM treatment (Fig. 7c).

## Discussion

In the present work, we tested the hypothesis that physiological saline solution-supplemented CM protects vascular grafts of BD rats from IR injury. To the best of our knowledge, this is the first study showing that the preservation of the aortic rings from BD rats with CM alleviates endothelial dysfunction following in vitro IR injury. CM has a preventive effect against endothelial dysfunction by lowering inflammatory response (through VCAM-1, ICAM-1 expression regression) and reducing caspase-mediated apoptosis.

Brain death in organ donors is a dynamic process with the following mechanisms associated with endothelial dysfunction: (a) the rapid rise of catecholamines, which inactivates endothelial relaxing substances (e.g., nitric oxide) and induces endothelin-1, a potent endothelium-derived contractile factor [31]; (b) hormone depletion, especially the decrease of thyroid hormones, which also results in impaired endothelium-dependent vasodilation [32]; (c) inflammatory cascade caused by endothelial activation after brain death [33]; and (d) the occurrence of oxidative stress, as well as apoptosis and nitro-oxidative stress-induced endothelial cell damage during brain death [34, 35]. In the current study, we confirmed that brain death was associated with impaired hemodynamic parameters [26, 27] and showed vascular functional alterations (decreased endothelium-dependent vasorelaxation to ACh, increased contractile responses to both PE and high K^+^-induced depolarization). BD-induced inflammation [36] may result in functional changes, as evidenced in the present study by elevation of MPO positivity in line with neutrophil infiltration. Furthermore, excessive generation of ROS and subsequent nitro-oxidative stress have been implicated in tissue injury following brain death. We showed that brain death led to increased formation of nitrotyrosine, a biomarker of nitrosative modification of proteins. Additionally, apoptosis can be triggered by three main signaling pathways, including extrinsic (death receptors) and intrinsic (mitochondria or the endoplasmic reticulum) pathways. We demonstrated that brain death-induced vascular functional alterations were also associated with caspase-mediated apoptosis, shown by increased caspase-3, caspase-8, caspase-9, and caspase-12 immunoreactivity, leading to excessive programmed cell death.

On the other hand, IR injury is an inevitable process during organ transplantation, which causes endothelial damage. Interestingly, even though IR injury impaired relaxant and contractile responses after brain death, the combination had no further effect on already high levels of MPO, nitrotyrosine, caspase-3, caspase-8, caspase-9, and caspase-12. In transplantation, currently, hearts are usually obtained from BD donors; however, more than a quarter of these potential donors must be excluded from transplantation due to ventricular dysfunction and contractility failure [37]. Therefore, new therapeutic strategies are required for protecting the endothelium against oxidative stress, apoptosis, and inflammation, as seen in brain death and IR injury, to optimize graft preservation and potentially increase the donor pool. In the present study, the preservation of aortic rings from BD rats with CM improved impaired endothelium-dependent vasorelaxation to ACh following IR injury. Bone marrow-derived MSCs secrete many known mediators, including chemokines, cytokines, growth factors, and others that contribute to tissue protection. In the present work, among the 39 proteins identified in CM, some of them, such as tissue inhibitor of metalloproteinase (TIMP)-1, growth hormone, prokineticin, vascular endothelial growth factor (VEGF), and activin A factors, are potential mediators which may confer protection and improve functional outcome after vascular IR injury. It has been shown that VEGF regulates endothelial cell survival, and the inhibition of apoptosis may represent a major aspect of the regulatory activity of VEGF on the vascular endothelium [38]. Furthermore, in isolated hearts, coronary perfusion pressure alterations in response to ACh have been demonstrated in growth hormone-deficient rats [39].

### Mechanisms underlying the vascular protective effects of CM after IR injury when using vascular grafts from BD donors

Coronary endothelial dysfunction has been described following brain death [3] and during cardiac transplantation-induced IR injury [40]. Inflammation, oxidative and nitrosative stress, and apoptosis-mediated cell death are the main responsible pathological factors. Enhanced ROS generation and activated neutrophils leading to apoptosis occur during brain death and cold ischemia.

Cellular damage induced by ischemia and aggravated by reperfusion activates an extensive inflammatory response. In the present study, the preservation of the aortic rings from BD rats with CM decreased the level of MPO following IR injury, the most abundant pro-inflammatory enzyme released upon neutrophil activation. Furthermore, increased gene expression of vascular inflammation-related specific adhesion molecules ICAM-1 and VCAM-1 following IR injury, reflecting endothelial dysfunction, has been lessened by CM.

The activation and adhesion of neutrophils to the endothelium is followed by a release of pro-inflammatory agents and by the production of excessive reactive nitrogen species and ROS. Mihm et al. showed that high nitrotyrosine concentrations, as a molecular footprint of peroxynitrite, may contribute to vascular endothelial dysfunction and concentration-dependent impairment of ACh maximal response [41]. Endothelial function can be assessed by the analysis of endothelial cells’ responsiveness to the vasodilator ACh. In line with these observations, the preservation of BD-IR aortic rings with CM significantly improved impaired ACh-induced endothelium-dependent vasorelaxation and showed a tendency to decrease nitrotyrosine immunoreactivity. In a rat model of diabetes mellitus-associated vascular endothelial dysfunction, MSCs-CM perfusion was shown to ameliorate compromised aortic vasodilation and alleviate oxidative stress in the aortas [42].

Another mechanism of reperfusion-linked tissue injury is apoptosis. Caspase-8, caspase-9, and caspase-12 are crucial molecules of three apoptosis pathways, namely, death receptors-mediated extrinsic pathway, intrinsic pathway, and endoplasmic reticulum stress-mediated pathway, respectively, which subsequently activate caspase-3. The decreased caspase-3, caspase-8, and caspase-9 immunoreactivity showed that CM reduced apoptosis in the aorta injured by brain death and IR injury. In line with these results, the mRNA caspase-3 levels, the main downstream effector of caspase, showed a decreasing trend after the preservation of aortic rings with CM. TIMPs are inhibitors of matrix metalloproteinase (MMPs) but also have MMP-independent functions. It has been shown that exogenous TIMP-1 prevents endothelial cell apoptosis through the activation of cell survival pathways [43]. Furthermore, the aorta of endothelial-specific prokineticin receptor-1 deficient mice displayed progressive impairment of ACh mediated endothelium-dependent relaxation, because of decreased nitric oxide synthesis [44]. It should also be mentioned that both pro-apoptotic and anti-apoptotic proteins were detected in the CM. We believe that the balance between pro- and anti-apoptotic factors in CM solution ensures tissue homeostasis, and its disruption may have adverse effects. It has also been reported that CM from multipotent stromal cells inhibits hypoxia-induced apoptosis and increases the survival of human aortic endothelial cells [45].

Even though we could not provide direct mechanistic evidence that the improved endothelial function was due to secreted factors by CM, our study did show that neutrophil infiltration was attenuated; immunoreactivity against caspase-3, caspase-8, and caspase-9 proteins was decreased; and ICAM-1 and VCAM-1 gene expression had regressed in vascular grafts from BD animals submitted to IR injury. In line with previous studies, our data suggest that the “cocktail” of several soluble factors secreted by bone marrow-derived MSCs acting together may contribute to tissue protection, thereby improving vascular graft function after IR injury. Further experimental research should be designed to elucidate the role of other factors identified in MSCs-CM.

There are some limitations to our study. Firstly, it would have been more clinically relevant to investigate the effect of CM on coronary endothelial dysfunction after IR injury than on the aorta; however, this is due to size limitations. However, the dimension and structure of the rat thoracic aorta are close to the large mammals and to human coronary arteries. Secondly, our ex vivo vascular ring apparatus examines the vascular reactivity but did not involve non-aortic tissues, blood flow to the tissues, and activation of leukocytes, which is required to be translated into a clinically relevant in vivo situation. Thirdly, identification of key candidate proteins detected in CM would help to elucidate the mechanisms involved in the protective effect. However, we have no direct evidence at this point. Finally, it remains unclear if other pathways play an essential role in the beneficial effect of CM on vascular graft from BD rats submitted to IR injury.

## Conclusions

In conclusion, the present study reveals that MSCs-CM significantly attenuates endothelial dysfunction against IR injury in the aorta of BD rats, in part, by lowering inflammatory response (through VCAM-1, ICAM-1 expression regression) and reducing caspase-mediated apoptosis. These likely do not represent the sole mechanism responsible for CM-mediated protection and further investigation is required to provide other possible mechanisms. From the clinical point of view, if hypothermic organ preservation could be improved, higher protection against both IR injury and brain death would be obtained, and the risk of posttransplant organ dysfunction would be reduced.

## Supplementary Information

**Additional file 1.** : Online Table 1: Sequence of primers for real-time PCR and Universal Probe Library (UPL) probes.

## Data Availability

All data generated or analyzed during this study are included in this published article and its supplementary information files.

## Abbreviations

ACh: Acetylcholine
BD: Brain-dead
CM-MSCs: Conditioned medium from mesenchymal stem cells
D-MEM: Dulbecco’s modified Eagle’s medium
DPBS: Dulbecco’s phosphate-buffered saline
EC_50_: Concentration of substance that elicited 50% of the maximum response
ICAM-1: Intercellular adhesion molecule-1
IR: Ischemia/reperfusion
KCl: Potassium chloride
KH_2_PO_4_: Potassium dihydrogen phosphate
KHL: Krebs-Henseleit
MMP: Matrix metalloproteinase
MPO: Myeloperoxidase
MSCs: Mesenchymal stem cells
NaCl: Sodium chloride
Na_2_HPO_4_: Sodium hydrogen phosphate
NaOH: Sodium hydroxide
pD_2_: -logEC50
PE: Phenylephrine
*R*_max_: Maximum endothelium-dependent relaxation
ROS: Reactive oxidative species
SEM: Standard error of the mean
SNP: Sodium nitroprusside
TIMP-1: Tissue inhibitor of metalloproteinase-1
VCAM-1: Vascular cell adhesion molecule-1
VEGF: Vascular endothelial growth factor
