# Supplementary material for: Mesenchymal stem cell-derived conditioned medium protects vascular grafts of brain-dead rats against in vitro ischemia/reperfusion injury
Source: Stem Cell Res Ther. 2021 Feb 24;12:144. doi: 10.1186/s13287-021-02166-3 (PMC7905634; doi:10.1186/s13287-021-02166-3)
Supplement: Supplementary file 1 — Additional file 1. : Online Table 1: Sequence of primers for real-time PCR and Universal Probe Library (UPL) probes. [file 13287_2021_2166_MOESM1_ESM.docx]

**Online Table 1: Sequence of primers for real time PCR and Universal Probe Library (UPL) probes.**

| Gene | Forward (F) and reverse (R) primer | UPL probes |
| --- | --- | --- |
| Caspase-3 | F:5’-CATgACCCgTCCCTTgAA-3’  R:5’-CCgACTTCCTgTATgCTTACTCTA- 3’ | 5 |
| ICAM-1 | F:5’-TCCAgCTCCACTCgCTCT-3’  R:5’-gCAgACCACTgTgCTTTgAg- 3’ | 29 |
| VCAM-1 | F:5’-ggTTCTTTCggAgCAACg-3’  R:5’-CAAATggAgTCTgAACCCAAA - 3’ | 13 |
| GAPDH | F:5’- CTACCCACGGCAAGTTCAAT -3’  R: 5’- ATTTGATGTTAGCGGGATCG -3’ | 111/9 |

ICAM-1 indicates intercellular adhesion molecule, VCAM-1 vascular cell adhesion molecule, and glyceraldehyde 3-phosphate dehydrogenase (GAPDH).
